# Deer antler renewal gives insights into mammalian epimorphic regeneration

**DOI:** 10.1186/s13619-023-00169-4

**Published:** 2023-07-25

**Authors:** Chunyi Li

**Affiliations:** 1 Institute of Antler Science and Product Technology, Changchun Sci-Tech University, Changchun, 130600 China; 2 Jilin Provincial Key Laboratory of Deer Antler Biology, Changchun, 130600 China; 3 College of Chinese Medicinal Materials, Jilin Agricultural University, Changchun, 130000 China

**Keywords:** Antler, Antler regeneration, Epimorphic regeneration, Digit/limb stump, Bone fracture, Callus

## Abstract

Deer antlers are the only known mammalian organ that, once lost, can fully grow back naturally. Hence, the antler offers a unique opportunity to learn how nature has solved the problem of mammalian epimorphic regeneration (EpR). Comprehensive comparisons amongst different types of EpR reveal that antler renewal is fundamentally different from that in lower vertebrates such as regeneration of the newt limb. Surprisingly, antler renewal is comparable to wound healing over a stump of regeneration-incompetent digit/limb, bone fracture repair, and to a lesser extent to digit tip regeneration in mammals. Common to all these mammalian cases of reaction to the amputation/mechanical trauma is the response of the periosteal cells at the distal end/injury site with formation of a circumferential cartilaginous callus (CCC). Interestingly, whether the CCC can proceed to the next stage to transform to a blastema fully depends on the presence of an interactive partner. The actual form of the partner can vary in different cases with the nail organ in digit tip EpR, the opposing callus in bone fracture repair, and the closely associated enveloping skin in antler regeneration. Due to absence of such an interactive partner, the CCC of a mouse/rat digit/limb stump becomes involuted gradually. Based on these discoveries, we created an interactive partner for the rat digit/limb stump through surgically removal of the interposing layers of loose connective tissue and muscle between the resultant CCC and the enveloping skin after amputation and by forcefully bonding two tissue types tightly together. In so doing partial regeneration of the limb stump occurred. In summary, if EpR in humans is to be realized, then I envisage that it would be more likely in a manner akin to antler regeneration rather to that of lower vertebrates such as newt limbs.

## Background

Growing back lost organs/appendages, a process known as epimorphic regeneration (EpR), in humans is the “Holy grail” of modern regenerative medicine, which is sustained by different animal model systems (Goss 1983; Stocum 2006; Carlson 2007). Models of mammalian EpR are rare but are highly desirable if successful strategies are to be devised for the restoration of damaged organs or limbs of humans. Currently, the most popular mammalian EpR model is regeneration of the digit tip, where following the loss/amputation of the distal region of the terminal phalanx (P3) in a mouse or human, blastema formation ensues and the lost part is restored (Neufeld and Zhao 1995; Muneoka et al. 2008; Storer and Miller 2020); however, this model is very simple and limited in regeneration potential. The most spectacular model for mammalian EpR is the annual renewal of large appendages (more than a meter long), the deer antlers, in which antlers not only fully regenerate with the complex species-specific morphology, but do so repeatedly with a growth rate reaching up to 2 cm/day. Despite this, study of antler regeneration has been largely neglected in the field of EpR regeneration.

## Antler regeneration

### Morphogenesis and histogenesis 

Regeneration of deer antlers takes place in a well-defined yearly cycle: in most species, in spring the previous hard antler (calcified bone) is cast from the permanent bony protuberance or pedicle; the stump wound heals rapidly and wound healing is followed by the commencement of new (soft) antler regeneration; there follows in late spring and early summer, a period of rapid elongation (up to 2 cm/day); at this stage, the antler is wrapped with special pelage, known as the “velvet skin”; in late summer/early autumn, the process of calcification starts to accelerate proximal-distally, blood supply to the velvet skin ceases and the velvet is shed to expose hard bony antlers for the rut (mating season); the hard antler is retained over winter and cast in the next spring to trigger a new round of antler regeneration (Goss 1983; Kierdorf et al. 2009; Li and Chu 2016).

**Fig. 1:**
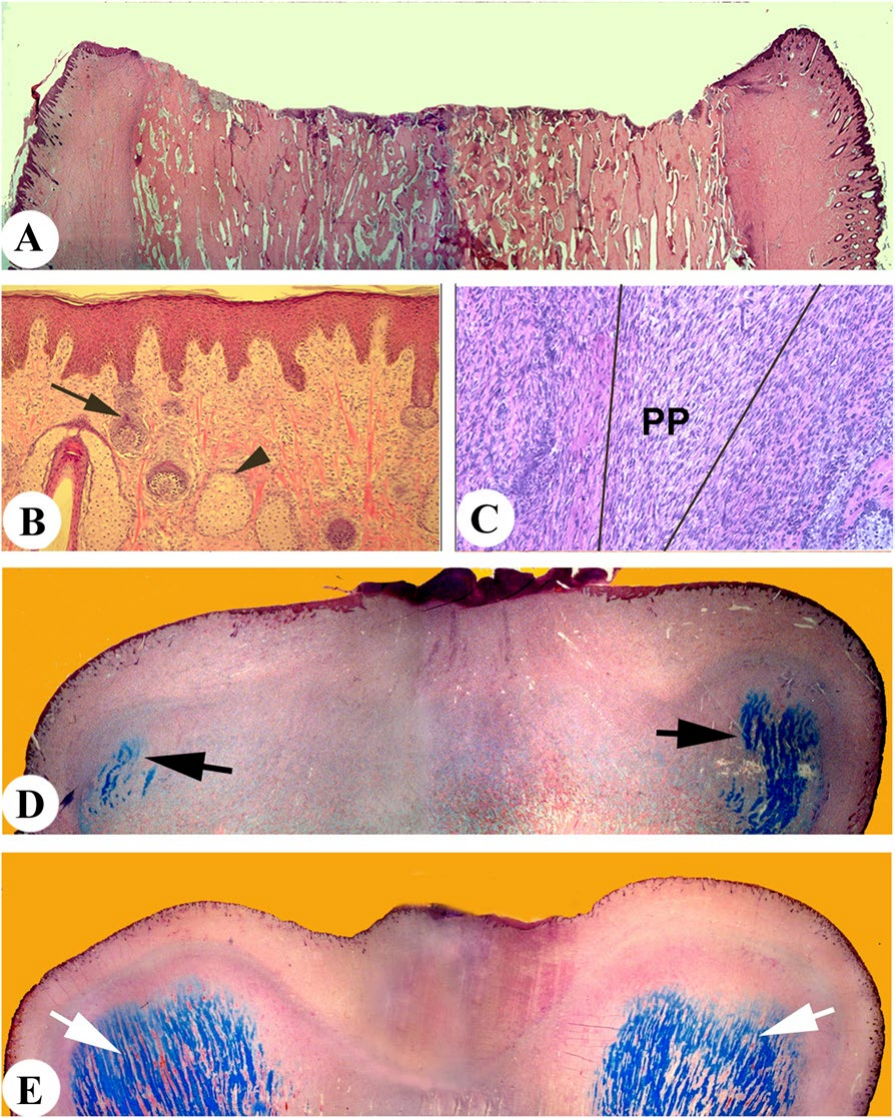
Histogenesis of a regenerating antler bud. **A** Sagittally-cut histological section of a pedicle stump immediately after hard antler casting; note the rough casting surface. **B** Epidermis of the skin rim formed by distal pedicle skin; note that this epidermis became thickened and acquired some velvet skin features. **C** The thickened hyperplastic perichondrium formed directly by distal pedicle periosteum (PP). **D** Sagittally-cut section of an early regenerating antler bud over a pedicle stump; note that circumferential cartilaginous callus (CCC) had formed at the anterior and posterior sites (two arrows). **E** Sagittally-cut section of a regenerating antler bud; note that rapidly-accumulating tissue mass in each CCC had pushed anterior and posterior corners laterally and distally (two arrows), and that the posterior and anterior bulges were the growth centres for the formation of the main beam and brow tine of the antler. Arrow: developing hair follicles; Arrowhead: sebaceous glands. PP: pedicle periosteum

Immediately after a hard antler is cast, the centre of the pedicle stump (Fig. 1A) is surrounded by a rim of shiny skin with very sparse hairs, being typical characteristics of velvet skin (Fig. 1B). Distal pedicle periosteum (PP), a tissue that is closely attached to the rim of this shiny skin, thickens through the active proliferation of cells resident within it (Fig. 1C). Subsequently, at the late wound healing stage, two crescent-shaped growth centres are formed at the distal end of a pedicle stump directly from the thickening PP, one located anteriorly and the other posteriorly. Each centre is made up of cartilaginous clusters that are capped by a layer of hyperplastic pedicle periosteum/perichondrium (Fig. 1D). Further augmentation of each growth centre raises the anterior and posterior portions of the pedicle stump more laterally and less distally at the early stage, although at the late stage the prominently protruded growth centres start to go beyond the cast plane distally and leave the central scab region behind (Fig. 1E). These posterior and anterior growth centres are the centres for the formation of the antler “main beam” and the “brow tine”, respectively. It has not been reported how a primitive deer species, such as roe deer and muntjac, regenerates their antlers with their brow tines quite distance above their burr. Nonetheless, it seems at the morphological (Li et al. 2004b) and the histological (Li et al. 2005) levels that it is the PP that gives rise to regenerating antlers.

### Discovery of the tissue and cell type for antler regeneration 

**Fig. 2:**
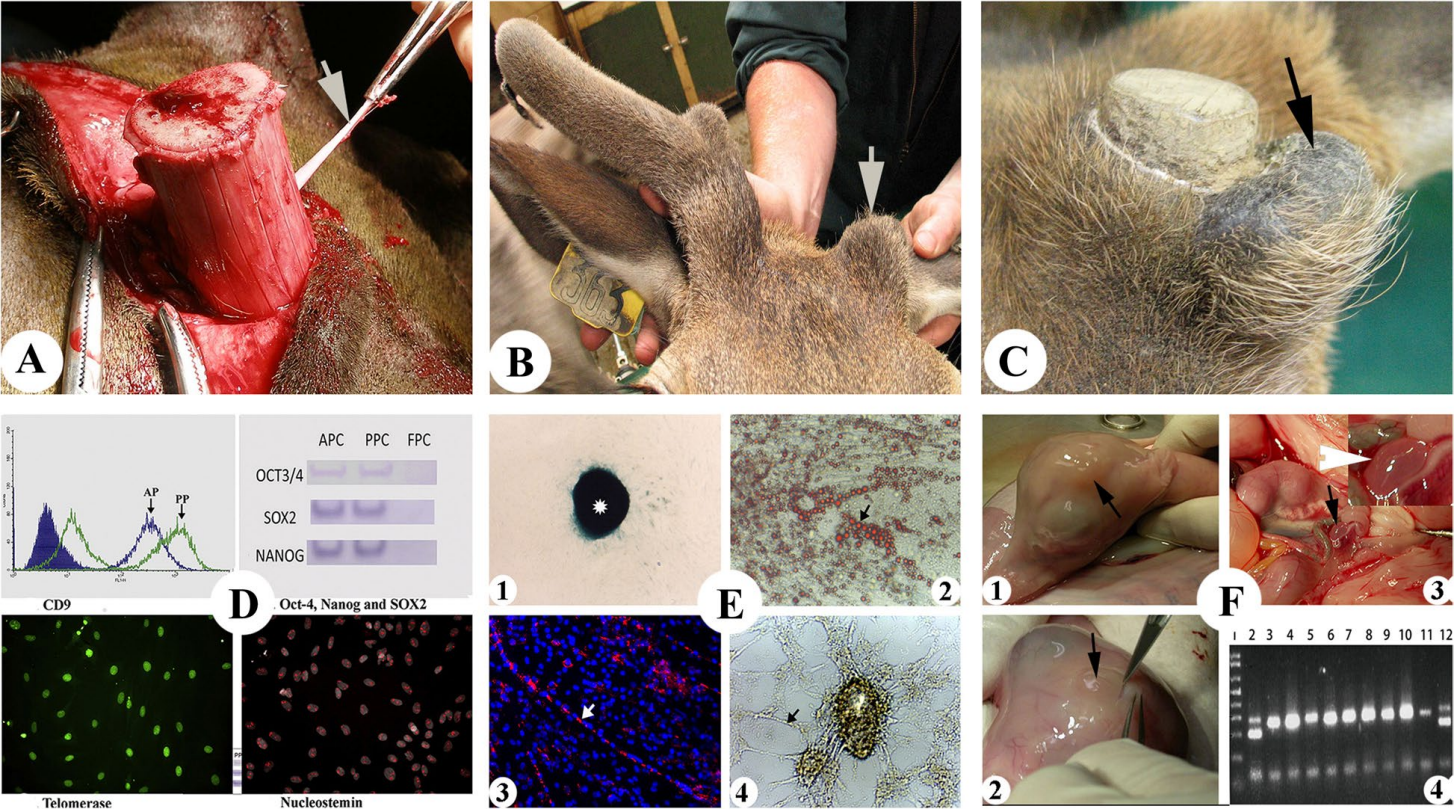
Identification of the tissue and cell types for antler regeneration. **A** Deletion of the pedicle periosteum (PP; arrow) prior to antler regeneration. **B** The PP-less pedicle failed to regenerate antler (arrow), whereas the sham-operated pedicle gave rise to a 3-branched antler. **C** Antler regeneration took place from the cut-end of PP on the pedicle bone shaft (arrow) when the distal third of PP was deleted. **D** Expression of key embryonic stem cell markers of the PP cells: CD9, Oct4, Nanog, SOX2, TERT and nucleostemin. **E** PP cells were induced to differentiate into different lineage cells: chondrocytes (E1), adipocytes (E2), myotubes (E3) and neuronal-like cells (E4). **F** Antlerogenic periosteal cells, from which PP cells were directly differentiated, were injected into the inner cell mass of female deer blastocysts; note that the resultant female fetuses developed primordial pedicles (F1 and F2), and that one animal also developed a testis (F3 and inset of F3) and this was confirmed to having been derived from the injected deer cells (F4). APC: antlerogenic periosteal cells; PPC: pedicle periosteal cells; and FPC: facial periosteal cells

Mindful of the perils of defining a dynamic process of antler regeneration based solely on a static histological description, we carried out functional analysis via PP deletion experiments. Complete removal of the PP (Fig. 2A) abrogates antler regeneration (Fig. 2B), whereas partial deletion of the PP (distal third) results in an antler that regenerates from the cut-end of the residual PP on the pedicle shaft (Fig. 2C), which is distant from the pedicle cast plane where the antler regenerates naturally (Li et al. 2007a). Therefore, it is the proliferation and differentiation of the PP cells that result in the regeneration of antlers, and no dedifferentiation process is observed during the initial stage of antler regeneration (Li 2013). We have estimated that around 3.3 million PP cells participate in each round of antler regeneration in red deer and give rise to up to 15 kg antler tissue mass within 70 days (Li et al. 2009).

Attributes of the PP cells seem extraordinary. We have then characterized these cells and found that they express both adult mesenchymal stem cell markers such as CD73, CD90 and CD105 (Wang et al. 2019) and some key embryonic stem cell markers, such as Oct4, Nanog and SOX2, TERT and nucleostemin (Fig. 2D; Li et al. 2009); these cells are capable of self-renewal and can be induced to differentiate into multi cell lineages, such as chondrocytes, osteoblasts, adipocytes, myotubes and neuronal-like cells (Fig. 2E; Li et al. 2009), Interestingly, when male periosteal cells were injected into the inner cell mass of female blastocysts, the female fetuses that developed pedicle primordia (Fig. 2F1 and F2; Wang et al. 2019). Most surprisingly, one of the female fetuses developed a testis (Fig. 2F3), which was found to have differentiated from the injected periosteal cells (Fig. 2F4). Therefore, we concluded that PP cells are an intermediate cell type between adult and embryonic stem cells and termed antler stem cells (Li et al. 2009).

## Antler regeneration vs EpR in lower vertebrates

The apparent similarities between regeneration of antlers and amphibian limbs (such as newts), which is the gold standard for the classical blastema-based EpR, has prompted some biologists, such as Goss (1983; 1980; 1985; 1992), to suggest that regeneration of antlers is realized through the same mechanism as that operating in lower vertebrates. However, some researchers recently considered that antler regeneration differs fundamentally from limb regeneration in urodeles (Kierdorf et al. 2009; Li et al. 2009); but antler regrowth was a form of epimorphic regeneration in vertebrates (Kierdorf and Kierdorf 2012). Because blastema formation is the hallmark of EpR, this mode of regeneration is also referred to as a “blastema-based” process. A blastema has been classically defined as the cone-shaped mass of dedifferentiated cells of diverse origins remaining on a stump after amputation of an appendage (Goss 1983; Meschaks and Nordkvist 1962; Mescher 1996). Blastema formation is considered diagnostic of EpR.

Tassava and Olsen (1982) stated that to realize EpR, three elements must be met: (1) wounding—they noted that in the absence of injury or amputation, it could not be called regeneration as nothing lost is to be replaced, cell dedifferentiation does not occur and therefore no potentially cycling cells are available for regeneration; (2) nerve input—without nerves, the cells exhibit very limited/no mitosis; and (3) wound epidermis—in the absence of a wound epidermis, dedifferentiated cells do not remain in the cycling state – that is, they either become arrested somewhere in the cell cycle, probably G1, or they leave the cell cycle and redifferentiate into other tissues (Globus et al. 1980; Loyd and Tassava 1980; Mescher 1976). In this respect, dedifferentiated cells must pass through sufficient numbers of cycles to provide a large enough population for EpR to occur (Loyd and Tassava 1980; Mescher and Tassava 1975; Salley and Tassava 1981; Tassava and Mescher 1975). Therefore, the cell proliferation potential of the distal end of a stump is the key to the realization of EpR.

### Wounding

Initiation of EpR, like limb regeneration, requires a mechanical trauma, such as amputation (Stocum 2006). Therefore, the question arises as to whether antler regeneration requires mechanical wounding? The answer is both yes and no. In most deer species, regeneration of the new antler closely follows casting of the old antler (Li et al. 2004b; 2005), a phenomenon that is superficially in line with the conclusion that mechanical trauma activates antler regeneration—that is, old antler casting (trauma) triggers new antler regeneration. Therefore, antler regeneration does seem to require mechanical wounding, but there are some exceptions to this general rule. For example, in some species, such as white-tailed deer, the old antlers are cast in winter, but the new antler growth is delayed until spring (Goss 1983); in fact, there is about a 3-month-gap between casting of the old antler and commencement of new antler regeneration. In reality, even in the deer species in which antler regeneration takes place immediately after hard antler casting, there are still some unanswered questions: new antlers still regenerate sometimes even when the old ones have failed to cast – in such cases, the antlers are of an abnormal shape (wrapping the hard antler base), such as natural “double-head” formation (Kierdorf and Kierdorf 1992) or artificially fixed the going-to-cast-hard-antler-base to the pedicle using a screw thus preventing casting (Goss 1983). Therefore, we conclude that the casting of the old antler and regeneration of the new antler are not causally related, although naturally the two processes follow one another in most deer species. This conclusion was also reached by Kierdorf and Kierdorf (1992). Logically, mechanical wounding of the pedicle may have been elicited at the time when antlers become totally calcified (i.e. dead) at the time of shedding of the velvet skin in the autumn, rather than at the time of hard antler casting in spring. Interestingly, such mechanical traumatization in autumn fails to trigger antler regeneration, which may further support the claim that wounding may not be a key requirement for antler regeneration. One may argue here that antlers fail to regenerate at the time of antler death probably because at that time hard antlers are in the way to effectively block the process. However, both that antler regeneration can still take place in the case of “double head” formation and antlers fail to regenerate in the 3-month-period of antler-less on top of the pedicle stump in white-tailed deer render this argument untenable.

If antler regeneration is not triggered by mechanical trauma, then what factor(s) might drive the initiation of antler regeneration? As the male secondary sexual character, antler regeneration is strictly under control of androgen hormones. Indeed, antler regeneration is triggered by the low threshold level of circulating androgen (particularly testosterone, T). For example, castration to a male deer at any time during hard antler phase, hard antlers will cast within two weeks and antler regeneration takes place immediately; whereas, administration of exogenous T will inhibit both hard antler casting and new antler regeneration relatively permanently (Akhtar et al. 2019; Bubenik 1982; Suttie et al. 1995a). Interestingly, in the case of castration of a male deer during the period of antler growth, the antlers continue to grow but the rate of growth gradually diminishes; so, no antler regeneration occurs, as nothing is lost to be replaced. To allow antler regeneration to take place in this situation, the pedicles must be re-primed by administration of high doses of androgen hormones to fully calcify the remaining antler tissue followed by withdrawal of androgens to induce the hard antler to cast. Therefore, wounding may potentiate the pedicle tissue, particularly the PP, and then the decrease in androgen level to certain threshold (< 0.5 ng/ml T) releases the “brake” to trigger the potentiated PP to regenerate the antler. In a sense, wounding in antler regeneration can be latent, in contrast to digit tip regeneration (narrow window response; Dawson et al. 2017).

Irrespective of wounding or androgen stimulation, there must be a way to generate potentially cycling cells for regeneration. That antler regeneration is coupled with androgen regulation may be advantageous for successful evolution of deer species.

### Nerve input

Farkas and Monaghan (2017) stated that nerve-dependency is the common phenomenon in the vertebrates that are capable of EpR. The typical case is salamander limb regeneration where blastema formation fails to occur in the absence of nerves (Singer 1946; Sugiura et al. 2016). The most convincing evidence of direct nerve-dependent effects of EpR is induction of limb regeneration by deviation of a transected nerve to a non-regenerative skin wound in the Axolotl (Satoh et al. 2007). Dependence on innervation has been attributed to the release by the nerves of factors that can stimulate blastema cell proliferation (Johnston et al. 2016; Rinkevich et al. 2014; Takeo et al. 2013).

Borgens (1982) reported that digit tip regeneration in rodents was also nerve-dependent, which extends the nerve-dependency claim to a wider spectrum of EpR. However, Simkin et al. (2015) found that denervated digit tips can undergo blastema formation and complete regeneration, and that the observed “nerve-dependent” phenomenon resulted from the decreased mechanical load to the leg as denervation of the sciatic nerve causes paralysis of the animal. Further analysis by Dolan et al. (2022) showed that denervated digits can undergo blastema formation and complete digit tip regeneration in the absence of peripheral innervation. However, the capacity for regeneration is attenuated in denervated digits, and this attenuation is attributed to an innervation-dependent delay in wound healing over an amputation stump. Therefore, the evidence is that innervation is not essential for successful regeneration of mammalian appendages, and that dependence on nerves in appendage regeneration is not a conserved vertebrate trait. Most importantly, in those mammalian systems which do exhibit regeneration, a nerve requirement has not been demonstrated; examples include holes that regenerate in denervated rabbit ears (Goss 1983) and in denervated wing membranes of the bat (Goss 1980).

Initially Bubenik (1982) believed that nerves are essential for antler regeneration and hypothesized an antler growth centre in the deer central nervous system (CNS). However, transection of the nerves to the pedicles in white-tailed deer (Wislocki and Singer 1946) and in red deer (Suttie and Fennessy 1985) did not affect subsequent antler regeneration, although it may have affected the antler size and shape to some extent. Even total denervation of the presumptive pedicle growth region on the frontal bone in prepubertal deer (sensory nerves (Li et al. 1993); or both sensory and sympathetic nerves (Suttie et al. 1995b)) carried out before pedicle and antler formation did not stop subsequent pedicle and antler formation or later antler regeneration. Therefore, nerve supply is not an indispensable requirement for antler regeneration. Goss (1995) considered that nerve input may only be required by those organs that require nerves to function, such as limbs. Further, the phenomenon of delays in wound healing caused by denervation (Dolan et al. 2022) is not observed in antler regeneration (Li et al. 1993). This is probably due to the fact that, apart from gravity, antlers/pedicles are not subject to mechanical load. From an evolutionary perspective, Dolan et al. (2022) found that EpR in different model animals varies considerably in terms of nerve- and/or mechanical load-dependency: regeneration in fish is both innervation and load dependent, regeneration in salamanders is innervation dependent but load independent, and regeneration in mammals (only few cases) is innervation independent and load dependent. Regeneration of deer antlers may provide the fourth category in that they are independent of both innervation and load. These contrasting situations are summarized in Table 1.

**Table 1:** Comparisons between a regenerating antler bud and a newt limb blastema

Antler bud	Limb blastema
Potentiated by natural loss of dead antlers, with regeneration activated when sex hormones reach a very low level	Regeneration activated by the accidental loss of distal part of a limb
Flat/concave shape	Round/cone shape
Formed by proliferation and differentiation of the PP cells	Formed by dedifferentiation, transdifferentiation and differentiation of diverse origin limb stump cells
Full thickness of skin heals the wound	Epithelium heals the initial wound
Presence of basal lamina	Absence of basal lamina
Richly vascularized	Avascular
Nerve-independent	Nerve-dependent
Wound epidermis-independent	Wound epidermis-dependent
Wound healing-independent	Wound healing-dependent
Healing with some evidence of a scar	Scar-less wound healing
Dividing cells regionally localized	Dividing cells evenly distributed

In considering other features relating to nerve input, Tassava and Olsen (1982) suggested that while nerves are very important for regeneration responses in lower vertebrates, there are little data to support this contention for higher vertebrates, and they proposed that the limiting factor for higher vertebrate limb regeneration may be the wound epidermis. In fact, failure of EpR in higher vertebrate limbs could be because the wound epidermis is nonfunctional and thus fails to allow cell redifferentiation to proceed.

### Wound epidermis

Tassava and Olsen (1982) concluded that the wound epidermis is absolutely essential for blastema formation; without wound epidermis, dedifferentiated cells do not remain in the cycling state and they arrest somewhere in the cell cycle, probably at G1/G0, or they leave the cell cycle and redifferentiate into other tissues (Globus et al. 1980; Loyd and Tassava 1980; Mescher 1976). Dedifferentiated cells must pass through sufficient cycles to provide a large cell population for EpR to occur, and this requires the presence of wound epidermis (Loyd and Tassava 1980; Mescher and Tassava 1975; Salley and Tassava 1981; Tassava and Mescher 1975). To determine how important the wound epidermis is for blastema formation, Goss and Holt (1992) amputated forelimbs of metamorphosed froglets (*Xenopus laevis*) to the wrist, skinned, and inserted them through the body wall into the abdominal cavity. In so doing, an epidermal wound was prevented and blastemas had failed to develop after two months, although the control limbs that were not inserted into the cavity formed a wound epidermis and the lost part was regenerated. Wound healing over a newt limb stump for blastema formation is a scar-less process and the basal lamina, a thin layer located between the dermis and the epidermis, is absent during formation of the blastema (Wallace 1981).

Interestingly, regeneration of antlers leaves a scar after the wound heals, albeit in most cases it is not obvious (Goss 1995) and a well-developed basal lamina is detectable in the healing skin over the pedicle stump (Li et al. 2004a; Kierdorf et al. 2007). Therefore, antler regeneration may not fully depend on the formation of wound epidermis. There is more convincing supporting evidence for this claim provided by our group, in which antler regeneration still took place even if the skin of a pedicle stump was physically prevented from participating in the wound healing process through inserting an impermeable membrane, although the regenerated antlers were skin-less and covered by a scab (Li et al. 2007b). Therefore, antler regeneration does not seem to rely on the presence of wound epidermis.

Typically, in non-regenerating limbs, there is a layer of connective tissue on the distal plane of an amputated stump that is interposed between the skin and underlying mesenchymal tissue. This tissue layer, known as the ‘pad’, ‘scar tissue’ or ‘dermal barrier’ forms not only in mammalian limb stumps but also in regeneration-incompetent frog limb stumps, in the limb stumps of fasted and hypophysectomized adult newts, and in denervated limb stumps of adult newts (Salley and Tassava 1981; Tassava 1969). Tassava and Olsen (1982) hypothesized that this tissue layer is the result, not the cause, of non-regeneration. However, in contrast, Goss (1995) considered that connective tissue layer interposed between the wound epidermis and the underlying mesenchymal tissues is the cause, not the consequence of the failure of EpR; and this layer may well constitute a barrier, both anatomically and physiologically, that interferes with whatever inductive communication might otherwise have taken place between these two important parts of a healing stump.

Irrespective of whether or not wounding is indispensable, and whether the wound epidermis and nerve input are essential for blastema formation and subsequent EpR, I believe that the functions of all these three key elements on blastema formation/EpR are to activate and sustain cell cycle progression in the initial regenerating buds. Tassava and Olsen (1982) also believe that there is no need for dedifferentiation in limbs of newborn mice and opossums, provided that there are cells that are sufficiently potent for proliferation present on the stump. That these undifferentiated cells continue to cycle after limb amputation is evidenced by the fact that the limb stumps increase in size and length. In this regard, antler regeneration does not seem to require all of these three seemingly indispensable elements, probably because the PP cells already possess almost unlimited potential for cell cycling, given that we have shown that around 3.3 million cells can form 15 kg of tissue mass within 70 days (Li et al. 2009).

## Antler regeneration vs digit tip EpR, stump healing and fracture repair

Comparative analysis shows that antler regeneration superficially resembles but, in fact, contrasts greatly with EpRs in lower vertebrates. Whether antler regeneration represents a unique phenomenon within the normal range of mammalian regeneration is not yet evident as there is a lack of comprehensive comparisons thus far. Therefore, here I consider similarities and contrasts of antler regeneration with three types of wound healing/regeneration, namely digit tip EpR, healing of a stump wound and fracture repair in mammals.

### Digit tip EpR

The EpR of the mouse digit tip is unique in that it occurs in a mammal, and thus provides a way to explore amputation injury responses that are either regeneration-competent or regeneration-incompetent. Therefore, digit tip regeneration currently serves as a popular mammalian model for EpR.

Amputation of the distal region of the terminal phalanx (P3) of mice causes an initial wound healing response followed by blastema formation and tip regeneration; in contrast, amputation at the proximal region of P3 and other digit segments, such as P2, fails to achieve EpR and results in bone truncation and soft tissue scar formation (Neufeld and Zhao 1995). Thus, digit regeneration is amputation level-dependent.

The reason why digit tip bone EpR is amputation level-dependent is due to the requirement for the presence of a nail organ; that is, EpR can only occur within the nail organ region (Neufeld and Zhao 1995; Han et al. 2008). Functional analysis has revealed that removal of the nail organ abrogated EpR of the digit tip amputated in the regeneration-competent region. In contrast, surgical retention of the nail organ stimulated EpR of the digit tip amputated in the regeneration-incompetent region (Zhao and Neufeld 1995; Mohammad et al. 1999). Takeo et al. (2013) reported that Wnt activation in the nail epithelium performs dual functions to promote both nail regeneration and Runx2^+^ mesenchymal cell growth through its ability to induce FGF2 expression. Thus, that amputation at the proximal region of P3 results in failure of EpR is because wounding at this level cannot activate epithelial Wnt signaling. Therefore, blastema formation for EpR of the mouse digit tip requires the interaction of blastema mesenchymal cells with nail organ epithelial cells.

Fernando et al. (2011) defined digit tip regeneration in mice through three stages: 1) a wound healing phase dominated by the extensive degradation of the stump bone, associated with enhanced osteoclast activities, prior to blastema formation; 2) the formation of a blastema with a reduced level of endothelial cells in conjunction with a reduced vasculature; and 3) an imprecise redifferentiation process via intramembranous ossification that produces the lost regenerates.

Epidermal closure during wound healing in the regeneration-competent P3 stump is a very slow process and is characterized by a failure of the epidermis to close across the amputated bone surface. Instead, the wound healing phase is associated with a strong osteoclast response that degrades the stump bone allowing the wound epidermis to undercut the distal bone resulting in a novel re-amputation response. Thus, this type of regeneration process initiates from a new level that is created by histolysis and proximal to the original plane of amputation. Fernando et al. (2011) considered that the extensive amount of bone erosion associated with adult digit tip regeneration provides a mechanism that exposes the bone marrow to the injury site allowing for the involvement of bone marrow-derived stem and/or progenitor cells in blastema formation. That termination of osteoclast activity is directly regulated by hypoxia supports this conclusion (Ji et al. 2015).

The blastema in the mouse digit tip EpR is an accumulation of undifferentiated cells, and there is evidence that the blastema itself is composed of a number of subpopulations of lineage-restricted cell types derived from different tissues of the stump (Lehoczky et al. 2011; Rinkevich et al. 2011; Takeo et al. 2013). These cells are possibly derived from a mixture of osteoprogenitor recruitment cells including periosteal cells (Loyd and Tassava 1980; Dawson et al. 2018; Lehoczky et al. 2011) and dedifferentiated mesenchymal cells (Storer et al. 2020; Johnson et al. 2020; Lehoczky et al. 2011).

The proliferating cells of the digit blastema express the mesenchymal cell marker vimentin and stem cell marker SCA-1 BMP4 (Han et al. 2008), and contain fewer endothelial cells than the surrounding tissue, indicative of reduced vascularity. Nerve-derived Schwann cells have also been shown to play a paracrine role in digit tip EpR by stimulating blastema cell proliferation (Johnston et al. 2016). For example, Lgr6 has been found to be expressed in nail stem cells of the digit and is required for digit tip EpR (Lehoczky and Tabin 2015). The dense central region of the blastema is avascular, hypoxic, is devoid of axons and Schwann cells, and is distinct from the peripheral connective tissues that are vascularized and contain non-myelinating Schwann cells (Dolan et al. 2019; Fernando et al. 2011; Sammarco et al. 2014; Yu et al. 2014).

Based on the descriptions of each regeneration process, it is evident that antler regeneration is quite different to that of digit tip EpR in the following ways: 1) the former experiences negligible histolysis, whereas the latter undergoes extensive osteoclastic activity and in so doing creates a new “amputation” cut; 2) formation of a blastema in the former is solely derived from proliferation and differentiation of the distal PP cells and a dedifferentiation process is not apparent, whereas in the latter, the process is partially through dedifferentiation; 3) the blastema/growth centre of the former is very richly vascularized and innervated, whereas the latter is relatively avascular, hypoxic and lacks neural input; 4) regeneration of the former is achieved via modified endochondral ossification, whereas the latter is via intramembranous ossification.

### Wound healing over the stump of a limb/digit

**Fig. 3:**
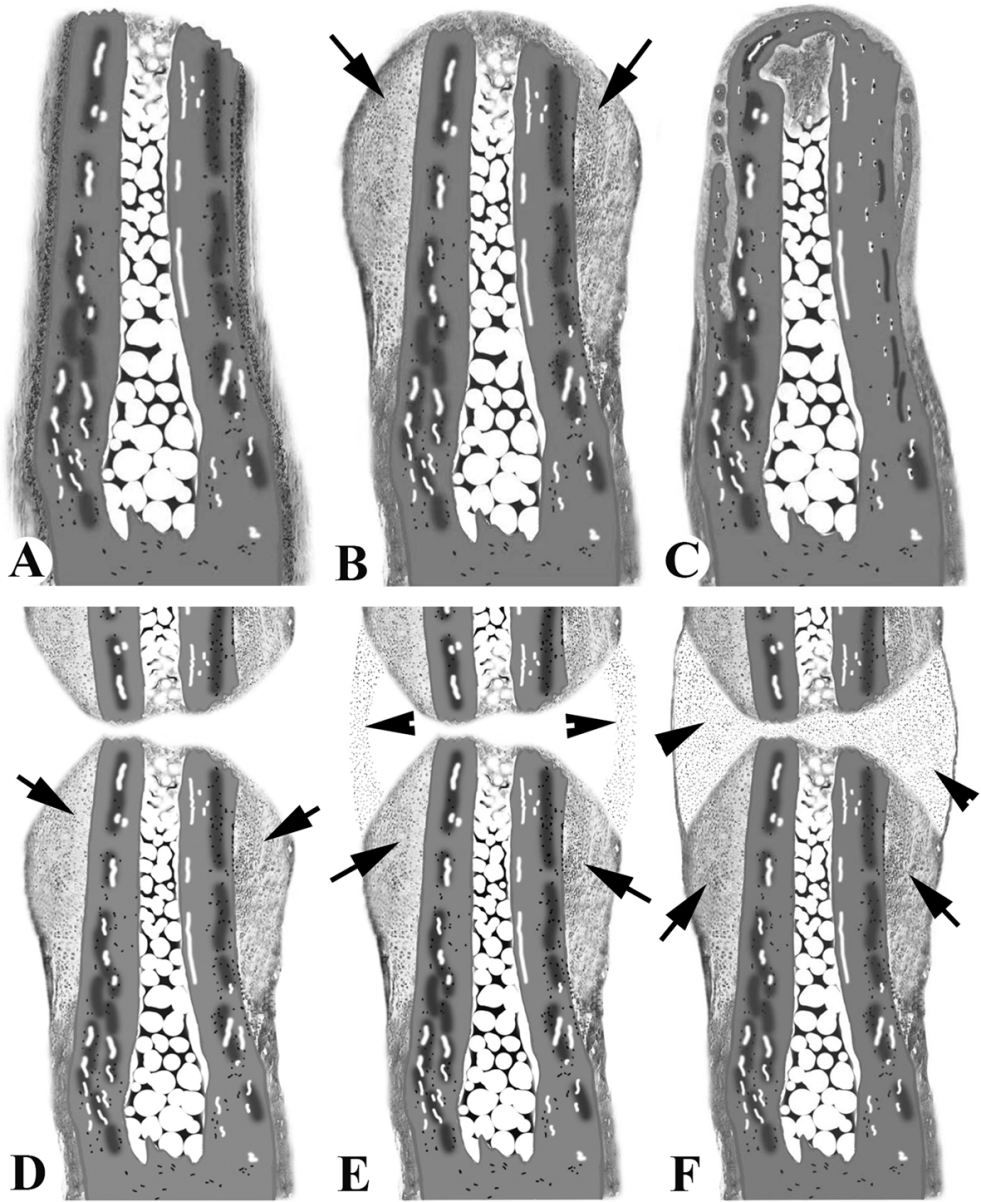
Schematics of stump wound healing process of second phalangeal element (P2; **A**-**C**) and fracture repair of P2 (**D**-**F**). **A** Freshly-amputated stump of a P2; note that the bone marrow cavity opens to the cut surface. **B** Prominent CCC (arrows) formed from the distal periosteal cells. **C** The CCC was remodeled to bone tissue; note that the bony callus gradually disappeared and eventually reverted to its original form. **D** Early stage of P2 fracture healing; note that CCCs (arrows) at both sides of the fracture line were formed. **E** Bridging callus (arrowheads) between the two CCCs started to form. **F** Late fracture healing stage; note that the bridging callus (arrowheads) had fully formed

Amputation of the middle phalanx (P2) or an area more proximal is regeneration-incompetent and is characterized by the formation of fibrous tissue capping the bone stump, a lack of distal bone growth and scar formation (Turner et al. 2010; Simkin et al. 2013; Agrawal et al. 2011a, 2011b; Mu et al. 2013). The anatomical changes in the P2 stump bone (Fig. 3A) indicate that the response to amputation injury is not static but dynamic (Dawson et al. 2018, 2017).

At 9 days post amputation (DPA), wound closure is complete and the healing epidermis is thickened in some regions over the P2 stump. The distal region of the bone marrow cavity is open to the amputation wound and the cavity itself is highly cellular. By this stage, a prominent chondrogenic callus has formed circumferentially, here termed the circumferential cartilaginous callus (CCC), around the lateral regions of the stump and this callus is formed by cells derived solely from the distal periosteum of the stump (Fig. 3B), and the osteoblasts that form the new bone are also derived from the periosteum. These findings have been functionally confirmed through periosteum deletion experiments. Histologically, at 9 DPA the periosteum-less bone stump failed to form a CCC, but the epidermal healing response and other tissues of the digit stump appeared normal (Yu et al. 2012; Dawson et al. 2016). By 15 DPA, the circumferential ossification of the stump bone had commenced. By 24 DPA, this ossification was apparent and a bone plug capped the stump, distally sealing the bone marrow cavity from the wound site, with the bone plug having formed by direct ossification. By 45 DPA, woven bone of the callus had been remodeled and the resulting bone more closely resembled the original bone (Fig. 3C). Therefore, formation of the CCC and subsequent transformation to woven bone are transient responses of the P2 bone to amputation injury; Dawson et al. (2017) considered that this response is analogous to a failed attempt at bone regeneration.

Overall, the wound healing process over a stump of regeneration-incompetent digit (P2 or more proximal fragments)/limb is very different to the blastema-based EpR of distal digit of P3 at levels of organ, tissue and cells, as the former has no regeneration beyond the amputation plane. Interestingly, antler regeneration and wound healing over a digit/limb stump are very alike, in that both form CCCs, derived solely from the proliferation and differentiation of the distal periosteal cells, and both of these calluses grow laterally but not distally passing the cast/amputation plane at the early stage. However, for some reasons, antler CCCs are able to go beyond this stage and to eventually regenerate antlers, but stump CCCs cannot (discussed in the next section).

Therefore, the general conclusion is that regenerative failure of P2 is causally linked to defects in the wound environment and is not limited by the availability of responsive cells. Using a GFP-label technique, Dawson et al. (2016) found that chondroprogenitor and osteoprogenitor cells of the periosteum participate in CCC formation following P2 digit amputation. Analysis of P2 bone stumps in which the endosteum and marrow are surgically removed but the periosteum is intact showed robust CCC formation. Overall, following P2 digit amputation, the bone stump undergoes an initial chondrogenic response by the periosteum that is followed by an ossification response which ultimately leads to an increase in stump bone volume. While digit amputation does not result in lengthening of the P2 bone (i.e. EpR), the stump tissue reacts to the injury by producing new bone tissue (i.e. tissue regeneration) that is organized circumferentially around the stump, i.e. the CCC. Therefore, I hypothesize that these periosteal cells would represent a target cell population for therapies aimed at enhancing the regenerative response/EpR following amputation.

### Wound healing of a long bone fracture

The healing of a fracture is one of the most remarkable repair processes in the body as it results in the actual reconstitution of the injured tissue resembling its original form. The repair of bone fractures is a postnatal regenerative process that recapitulates many of the ontological events of embryonic skeletal development (Einhorn and Gerstenfeld 2015).

The first evidence of increased cell division in the periosteum immediately around the fracture is to be found within about eight hours of the injury and reaches a maximum in about 24 h. At first, this activity extends throughout the whole length of the injured bone; however, within a few days, it declines and eventually becomes confined to the area immediately adjacent to the fracture where it remains above normal levels for several weeks (Tonna and Cronkite 1961). Interestingly, the broken bones do not themselves participate in this proliferative activity and repair, but are in fact dead, evidenced by the presence of empty osteocyte lacunae which are away from the fracture line for a variable distance (McKibbin 1978).

Fracture repair involves definition of specific morphogenetic fields and is thus dependent on interactions between various proximate tissues; the fracture line in the bone sets up the overall spatial relationships of the morphogenetic fields during tissue regeneration. This is shown by the development of two discrete CCCs, that are symmetrical with respect to the fracture line and taper proximally and distally along the cortices of the bone (Fig. 3D; Gerstenfeld et al. 2003; Gerstenfeld and Einhorn 2003).

The primary tissue source of stem cells that give rise to these CCCs are from the periosteum (Nakahara et al. 1990), evidenced by transgenic lineage tracking (Colnot 2009). This finding was convincingly confirmed by the functional analysis, in which the developmental capacity of these CCCs disappeared if the periosteum was removed at fracture (Buckwalter et al. 2001). In the first a few days and weeks, there develops what has been termed the primary callus (CCC); this response appears to be a fundamental reaction of the bone to injury and is almost independent of environmental circumstances. However, it is short-lived and disappears in the absence of contact with another fragment. It seems almost certain that the cells responsible for this activity arise from the bony tissues themselves, and particularly from the periosteum. Because this initial response is finite, bridging of the fragments cannot result from its activity alone; the next phase is formation of a bridging external callus (induced callus). This is a rapid process involving interactions between the fragments, particularly the two CCCs (Fig. 3E and F); this part of the process is known to be very dependent on mechanical factors (Gerstenfeld et al. 2003).

In comparison, the proximal bone fragment of a long bone fracture is reminiscent of a regeneration-incompetent limb/digit stump. While each forms a CCC, which is derived from the proliferation and differentiation of the periosteal cells, the differentiation fate of these two types of CCCs is quite different: the callus of the stump remodels to bone and gradually disappears back to its original form of the stump, calluses from both sides of the fracture line do not fade away, but instead a bridging callus gradually forms between them. Interestingly, McKibbin (1978) reported a case in which, due to an accident, a patient suffered a tibial fracture on one side while on the other side, they underwent amputation at approximately the same level. Six weeks later, both sides had formed CCCs, while the one on the proximal fragment of a fracture had started to form the bridging callus toward other side, CCC of the amputation stump was apparently inert even though it could be regarded as one side of a fracture. Clearly the response of this fragment is in some way dependent on the presence of its fellow. Interestingly, it has been found that formation of a bridging callus depends on the distance of the two CCCs between the fracture line (not too long not too short), indicating that it is the interaction between the two calluses that induces the formation of the bridging callus. Since formation of the bridging callus relies on the presence of the two partner CCCs, they were termed “osteogenic blastema” (Pritchard, 1978, which was cited by McKibbin 1978); or “fracture blastema” (Kellum et al. 2009).

The inductive substances responsible for bridging callus formation are not yet known. However, Bostrom et al. (1995) reported that the periosteal cells produce members of TGF-β superfamily during the initial healing phases of following fracture. BMP 2 and GDF 8 were maximally expressed on day 1, suggesting roles as early response genes in the cascade of healing events. GDF5, TGFb2, and TGFb3 showed maximal expression on day 7, when type II collagen expression peaked during cartilage formation. In contrast, BMPs 3, 4, 7, and 8 showed a restricted period of expression from days 14 through 21 at the time when the resorption of calcified cartilage and osteoblastic recruitment were most active. Yoshimura et al. (2001) reported that TGFb1, BMP5, BMP6 and GDF10 were constitutively expressed from days 3–21. On the other hand, the periosteal cells specifically react to BMPs in the early stages of response to fracture to promote both chondrogenesis and osteogenesis (Yu et al. 2010). Therefore, it is likely that the formation of a bridging callus relies on the exchange of BMPs between the two partner CCCs, which is further supported by the findings of Wang et al. (2011) through approach of BMP2 deletion (periosteal-derived BMP2 is required for induction of bridging chondrogenic callus after bone fracture).

If perfect bone fracture repair is achieved through exchange of BMPs between the two CCCs, the consequences of BMP2 application to the P2 stump would be of interest. Experimental results convincingly demonstrated that BMP2 treatment stimulated formation of a distal cartilaginous callus at the amputation site and completely restored the length of the P2 bone (Yu et al. 2012; Dawson et al. 2017). Therefore, the BMP family must function as a key endogenous factor controlling the periosteal response to injury irrespective of whether it is caused by an amputation or a fracture. In this regard, Dawson et al. (2017) found that treatment of a bone injury with BMP enhanced the endogenous response by extending the period of BMP2 signaling, thus effectively inducing regeneration. Surprisingly, the CCC of a pedicle stump in deer is no different to that of a digit/limb stump at tissue level, but the former seems itself endowed with full regeneration potential to regenerate antler in the absence of an interactive “third party”, such as an opposite callus, nor does it require the presence of an “inductive morphogen”, such as BMP2. Therefore, unveiling of this underlying unique mechanism may give insights into mammalian EpR, including humans. These similarities and contrasts are summarized in Table 2.

**Table 2:** Comparisons between antler regeneration and digit tip EpR, stump wound healing and fracture repair in mammals

Antler regeneration	Digit tip EpR	Stump wound healing	Fracture repair
Proliferation of periosteal cells to form a CCC	Extensive histolysis to release the cells for CCC and blastema formation	Proliferation of periosteal cells to form a CCC	Proliferation of periosteal cells to form CCCs
CCC interacts with the fused skin to initiate blastema formation and subsequent regeneration	Cell dedifferentiation and recruitment of progenitor cells; redifferentiation via intramembranous ossification	Fibrous tissue seals the open end of long bone, full thickness skin heals the wound, CCC remodels to bone and then disappears	Two CCCs interact with each other to initiate bridging callus formation and subsequent repair
Interactive partner: enveloping skin	Interactive partner: nail organ	Interactive partner: absence	Interactive partner: opposite callus
Interactive substances: ?	Interactive substances: Wnt factors	Interactive substances: BMP factors	Interactive substances: BMP factors
Growth via modified endochondral ossification	growth via intramembranous ossification	CCC formation via endochondral ossification	Both the CCCs and bridging callus via endochondral ossification
Both nerve and load independent	Nerve independent and load dependent	Nerve independent but load effect not known	Nerve independent and load dependent

## Exploration of the mechanism underlying full potential of pedicle stump regeneration

**Fig. 4:**
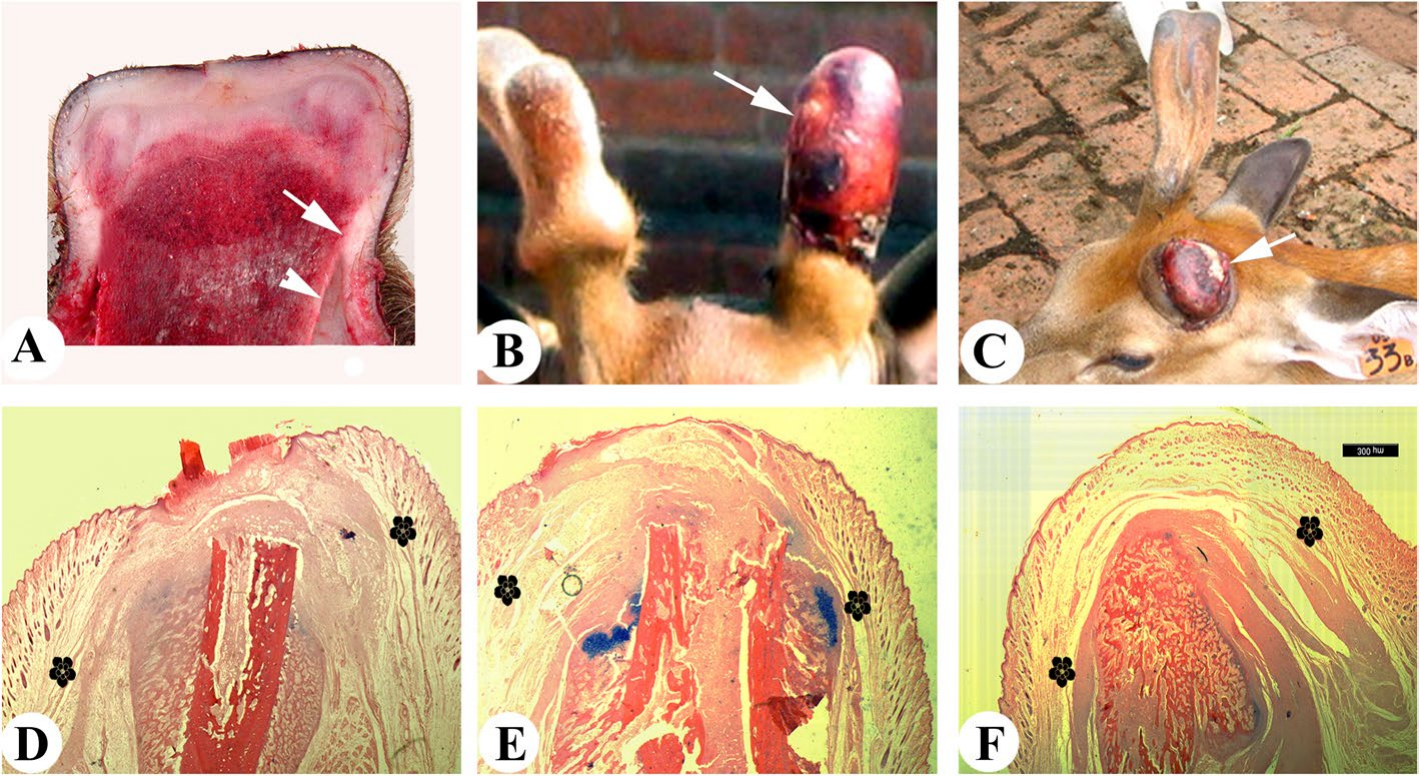
Relationship between the distal periosteum/CCC and the enveloping skin in both pedicle stump (**A**-**C**) and P2 stump (**D**-**F**). **A** Sagittally-cut surface at late wound healing stage; note that at distal third of the stump, the PP and the enveloping skin had almost fused together (white arrow), whereas at the proximal two-thirds, the two tissue types only loosely associated with each other (white arrowhead). **B** Skin-less antler (white arrow), regenerated after insertion of an impermeable membrane between the PP and the pedicle skin at the distal third (fused) of a pedicle stump prior to antler regeneration. **C** Laterally thickened pedicle stump (white arrow), created after insertion of an impermeable membrane between the PP and the pedicle skin at the proximal two-thirds region (loosely associated) of a pedicle stump prior to antler regeneration. **D** At an early wound healing stage of a mouse leg stump; note that the CCC had formed, but this callus was separated widely by layers of loose connective tissues and muscle (flowers). **E** At a late wound healing stage; note that the CCC and the enveloping skin were still widely separated by the multiple layers of tissues (flowers). **F** At the completion of wound healing; note that the open end of the bone marrow cavity had sealed, but the stump bone and enveloping skin were still widely separated by the multiple layers (flowers)

Observation of a longitudinal cut surface of a pedicle stump immediately after hard antler casting reveals that the degree of association between the PP and the enveloping skin varies considerably distal-proximally (Fig. 4A): over the distal third, the two tissue types are almost fused together (antler regeneration region), whereas at the proximal two-thirds, the two tissue types are only loosely associated (the non-antler regeneration region), suggesting that antler regeneration may depend on the interaction of the PP and the enveloping skin. Surprisingly, antler regeneration still takes place when pedicles shorten into the proximal loosely-associated region as deer age (each round of antler regeneration consumes a certain amount of pedicle tissue). Research finds that, by then, the PP and the skin have already become fused in this proximal region (Li 2013). Overall, these observations seem to support a conclusion that the intimate association between the PP and enveloping skin is indispensable for antler regeneration, i.e. the enveloping skin serves as the interactive partner to bestow the pedicle stump a full potential regeneration.

To functionally test this hypothesis, we inserted an impermeable membrane between the PP and the enveloping skin of a pedicle stump prior to antler regeneration (Li et al. 2007b). The results were astonishing: when the insertion site was at the fused region (distal third), the membrane effectively prohibited skin participation, but failed to inhibit antler regeneration; albeit the regenerated antler was skin-less and enclosed by a scab (Fig. 4B). When the membrane was inserted at the loosely-associated region (proximal two-thirds), antler regeneration failed to occur, although CCCs were formed (Fig. 4C). These experiments convincingly demonstrate that: 1) antler regeneration, but not CCC, relies on the interactions between the PP and the enveloping skin; 2) to enable the establishment of these interactions, the two tissue types must become intimately associated; and 3) these interactions are transient in nature as once the two interactive tissues become fused together, separation of pedicle skin from the PP can no longer prevent antler regeneration. Thus, antler regeneration can take place from the pedicle CCCs in the absence of the opposite callus because the PP has interacted with the enveloping skin, an alternative interactive partner. Identification of the putative interacting substances between the PP and the enveloping skin would greatly facilitate definition of the mechanism underlying antler regeneration as a unique model for mammalian EpR.

The reason why the distal periosteum/CCC of a digit/limb cannot interact with the enveloping skin to launch regeneration of the lost structure is subject to speculation. Interestingly, throughout the entire course of wound healing in a rat stump from the time of amputation to completion of healing, the distal periosteum/CCC and the enveloping skin are separated by multiple layers of muscle and loose connective tissues (Fig. 4D-F; unpublished). I hypothesized that it is these tissue layers that have effectively blocked the passage of interactive substances between the skin and the periosteum/CCC, resulting in failure of regeneration. Functional verification of this hypothesis would require surgical removal of these interposing barriers at the time of amputation and forcing of an intimate association between the two interactive tissue types (see next section).

## Antler regeneration provides insights into EpR of mammalian appendages

The ultimate goal of studying regeneration of antlers is not to satisfy one’s curiosity, but to learn whether the antler can be used as a suitable model for understanding EpR and its potential application in regenerative medicine. During evolution, mammals have largely lost the ability to replace their missing appendages (Wallace 1981). From the foregoing comparisons, it is evident that antler regeneration is fundamentally different from EpR in lower vertebrates and is even different from the EpR of mouse digit tips to some extent, but it is very similar to the early stage wound healing of the regeneration-incompetent digit/limb and of fracture repair in mammals. In EpR in lower vertebrates, a blastema forms through at least partial dedifferentiation and requires involvement of three key elements namely, wounding, a nerve input and a wound epidermis, in order to generate a sufficient number of cycling cells for blastema formation. However, in the case of the antler, a blastema forms through differentiation and does not need to experience these key elements as the PP cells already possess an extraordinary potential of multiplication. In the EpR of the mouse digit tip, the blastema forms through a combination of dedifferentiation and recruitment of resident progenitor cells, these cells having been acquired through extensive histolysis caused by osteoclastic activities over the stump. In contrast, the antler blastema is formed solely through proliferation and differentiation of the PP cells, dedifferentiation is not observed and only negligible osteoclastic activity is detected (Li 2013; Li et al. 2005). However, in a recent study (using single cell sequencing), we found that the blastemas of both the digit tip and antler, although formed through different routes, each contain a population of cells that exhibit similar profiles of gene expression, such as PTN, TNN, TNC and DLX5, whereas the blastema of the lower vertebrates do not contain this cell population (Qin et al. 2023). In the wound repair of a regeneration-incompetent digit/limb stump or of a fracture in mammals, the initial injury triggers proliferation and differentiation of the periosteal cells at the injury site and forms a CCC in each case. This is essentially the same process as in antler regeneration. Consequently, EpR in humans, if it is to be realized, would highly likely be through a means that is more akin to antler regeneration rather to that of lower vertebrates such as newts.

Through these comprehensive comparisons, blastema formation in mammalian EpR seems to undergo two stages: 1) CCC formation, and 2) blastema transformation from the CCC. The first stage is CCC formation through activation of proliferation and differentiation of the periosteum at the injury site and is common to all mammalian stumps of digit/limb including regeneration-competent digit tips and deer pedicles. This response is a reaction of the periosteum to injury and is virtually independent of environmental factors. However, it is short-lived and disappears if it does not progress to the next stage, namely formation of a blastema through interactions with a “third party”. The second stage of blastema formation from the CCC is highly environment-dependent but the interactive partners can vary. In the case of the digit tip EpR, a distal callus is formed mainly by the cells released through extensive histolysis, and the mesenchymal cells in the callus must interact with epithelial cells of the nail organ to be able to build up the blastema. In the case of fracture healing, the formation of a bridging callus (“the osteogenic blastema”), is achieved through interactions between the CCCs at two sides of the fracture line, possibly through exchange of BMPs. In the case of antler regeneration, the blastema is built up through interactions between the CCC and the intimately-associated enveloping skin, but the interactive substance/s are as yet unknown. In the regeneration-incompetent digit/limb stump, the “blastema” can be induced by topical application of BMP2 to the CCC before its involution, and this induced blastema has the potential to regenerate entire length of P2.

The current definition of the process of blastema formation has broadened the vision for EpR by including resident undifferentiated “stem cells” or progenitor cells to encompass examples of mammalian EpR (Bely and Sikes 2010). This is in contrast to the classical viewpoint, based on the amphibian EpR, where it was hypothesized that the process was solely dependent on cell dedifferentiation (Mescher 1996; Wallace 1981; Tsonis 2000; Goss 1969). However, whether a dedifferentiation-based (lower vertebrate) and a stem cell-based (mammal) blastema have a similar capacity to regenerate lost structures has not yet been addressed. In this respect, evidence to date suggests that the stem cell-based process is that which operates more in the regeneration of simpler systems, such as compensatory growth in response to increased functional load (such as removal of one kidney or partial hepatectomy; (Goss 1983; Stocum 2002)), whereas the dedifferentiation-based process is associated with the regeneration of more complex structures like organs and/or limbs. Li et al. (2014) provided a possible explanation for this claim in that a dedifferentiation-based process allows formation of a miniature prototype-structure of a lost part. This process complements that of developmental ontogeny, wherein a mini-organ, including joints, is developed at the initial stage, and then enlargement follows through growth to match the size of the organ that is lost. In contrast, a resident stem cell-based process builds up the missing structure through direct proliferation and differentiation of these cells, and as such, it may not be compatible with the formation of morphologically and structurally complex organs and/or appendages.

However, regeneration of deer antlers, which are morphologically complex appendages, is a residential stem cell-based process, and would seem to lead to a rejection of the above hypothesis, in that the encoded morphogenetic blueprint of species-specific antlers is unfolded as the appendage elongates. Despite the massive regenerative capabilities of antlers, it remains unclear as to whether such a process can cope with the regeneration of joints and muscles, as these are absent in antlers. In this respect, Yu et al. (2010) reported that BMP2 treatment of the P2 digit stump can stimulate EpR through inducing formation of a distal cartilaginous callus (osteogenic blastema), at the amputation site. This blastema can restore completely the entire P2 bone length, but not the joint or the distal P3 skeletal element. Therefore, this may reflect a limitation of the stem cell-based EpR.

Whether we can successfully induce EpR in the human through dedifferentiation-based blastema formation remains to be determined, but nature has solved the problem of regeneration of a mammalian appendage, the deer antler, through stem cell-based blastema formation, and so it offers a unique opportunity to learn from the nature. I believe that a new paradigm for successful mammalian regeneration is to understand how to create a blastema from the tissue-specific, lineage-restricted progenitor cells that have the ability to undergo individual tissue level repair, such as stump periosteal cells. If we can achieve partial regeneration in the clinical setting and if we can properly control and manage it, we would be able to further enhance the functionality for amputees and attain a better outcome beyond that of wound healing alone.

**Fig. 5:**
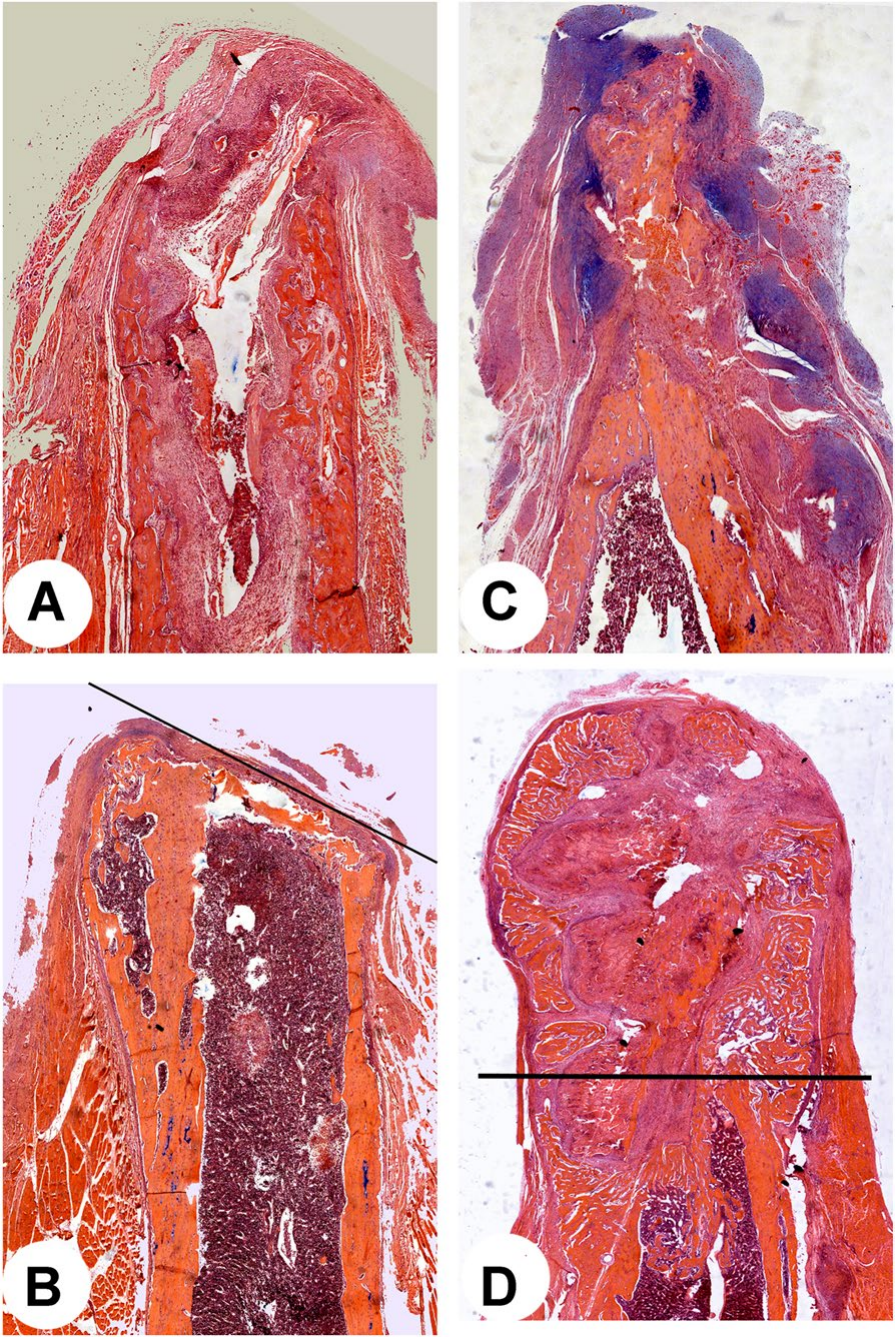
Longitudinal histology sections of rat leg stumps cut through mid-part of the front leg (Zhang et al., unpublished), which was then left untreated (**A** and **B**) or subjected to bioelectric stimulation to the stump periosteum (**C**) and removal of the interposing layers between the periosteum and the enveloping skin (**D**). Note that the stump that was stimulated by bioelectricity to the periosteum immediately after amputation showed discrete blue color dots (alcian blue staining), which are the attachment sites for electrodes. Two months after the treatment, the treated stump partially regenerated the lost leg, and the regenerate length is significantly longer than its width. The black line denotes amputation plane

To achieve the goal of EpR of a digit/limb stump, a proper partner must be created for the CCC of the stump to interact. Transplantation of a nail organ, although regeneration may be realized through a typical blastema, has only very limited growth capacity (maximally can regenerate a digit tip) and not event mention the availability of autologous nail organs. It is not practical to place another opposite interactive CCC for the stump, as it would a great challenge to hold this opposite CCC firmly with a finely-tuned distance between the two CCCs. Therefore, creation of the intimate association between the CCC and the enveloping skin seems to be the logical and practical choice. Along this reasoning, we recently developed a two-step-procedure to achieve partial EpR of the rat leg stump: 1) to enhance formation of the CCC, bioelectrical stimulation was applied to the distal periosteum following amputation trauma, and 2) to create an intimate association between the periosteum/CCC and the enveloping skin in order to facilitate their interaction, the interposing tissues including layers of loose connective tissue and muscle were removed surgically and the two interactive tissue types held tightly together (with a rubber band) after skin suturing. In so doing, blastema formation was induced successfully and partial stump regeneration ensued. Notably, the length of the regenerates was achieved significantly longer than their width (Fig. 5). Currently, we are seeking to deliver BMPs and/or the factors identified from the PP cells to the distal periosteum of the leg stump to further improve the quantity and quality of the regenerated limb.

In summary, a better understanding of the mechanisms that regulate the regeneration of antlers, the only mammalian organ that can fully and repeatedly regenerate, may provide valuable insights into the design of future treatment options in the rapidly evolving field of regenerative medicine.

## Data Availability

Not applicable.

## Abbreviations

PP: Pedicle periosteum
EpR: Epimorphic regeneration
CCC: Circumferential cartilaginous callus
P3: Terminal phalanx
P2: Phalanx 2
DPA: Days post amputation
DEGs: Differentially expressed genes
BMPs: Bone morphogenetic proteins
TGF-β: Transforming growth factor-β
GDF: Growth differentiation factor
PTN: Pleiotrophin
TNC: Tenascin C
TNN: Tenascin N
DLX5: Distal-less homeobox5
Oct4: POU class 5 homeobox 1
SOX2: SRY-Box Transcription Factor 2
